# Novel Terahertz Spectroscopy Technology for Crystallinity and Crystal Structure Analysis of Cellulose

**DOI:** 10.3390/polym13010006

**Published:** 2020-12-22

**Authors:** Rui Yang, Xianyin Dong, Gang Chen, Feng Lin, Zhenhua Huang, Maurizio Manzo, Haiyan Mao

**Affiliations:** 1 Co-Innovation Center of Efficient Processing and Utilization of Forest Resources, College of Materials Science and Engineering, Nanjing Forestry University, Nanjing 210037, China; yangrui@njfu.edu.cn (R.Y.); d549920@163.com (X.D.); cg13576431@163.com (G.C.); 2 Dehua Tubaobao New Decoration Material Co., Ltd., Huzhou 313200, China; 3 Advanced Analysis and Testing Center, Nanjing Forestry University, Nanjing 210037, China; ptfern@126.com; 4 Department of Mechanical Engineering, University of North Texas, Denton, TX 76207, USA; zhenhua.huang@unt.edu (Z.H.); Maurizio.Manzo@unt.edu (M.M.); 5 Department of Chemical and Biomolecular Engineering, University of California, Berkeley, CA 94720, USA; 6 Jiangsu Chenguang Coating Co., Ltd., Changzhou 213164, China

**Keywords:** terahertz spectroscopy, cellulose, nano-crystalline cellulose, crystalline

## Abstract

Crystallinity is an essential indicator for evaluating the quality of fiber materials. Terahertz spectroscopy technology has excellent penetrability, no harmful substances, and commendable detection capability of absorption characteristics. The terahertz spectroscopy technology has great application potential in the field of fiber material research, especially for the characterization of the crystallinity of cellulose. In this work, the absorption peak of wood cellulose, microcrystalline cellulose, wood nano cellulose, and cotton nano cellulose were probed in the terahertz band to calculate the crystallinity, and the result compared with XRD and FT-IR analysis. The vibration model of cellulose molecular motion was obtained by density functional theory. The results showed that the average length of wood cellulose (WC) single fiber was 300 μm. The microcrystalline cellulose (MCC) was bar-like, and the average length was 20 μm. The cotton cellulose nanofiber (C-CNF) was a single fibrous substance with a length of 50 μm, while the wood cellulose nanofiber (W-CNF) was with a length of 250 μm. The crystallinity of cellulose samples in THz was calculated as follows: 73% for WC, 78% for MCC, 85% for W-CNF, and 90% for C-CNF. The crystallinity values were obtained by the three methods which were different to some extent. The absorption peak of the terahertz spectra was most obvious when the samples thickness was 1 mm and mixed mass ratio of the polyethylene and cellulose was 1:1. The degree of crystallinity was proportional to the terahertz absorption coefficients of cellulose, the five-movement models of cellulose molecules corresponded to the five absorption peak positions of cellulose.

## 1. Introduction

Cellulose is a chain structure of large molecules and the crystallinity has a great influence in the field of fiber materials, such as mechanical properties, optical properties, thermal properties, etc. [1,2,3]. Nowadays, there are many methods to test the crystallinity of fibers, such as XRD and FT-IR, which detect the absorption peak characteristics of cellulose samples in a certain band [4,5,6]. However, the study of crystallinity at present is only in the medium and high frequency band. There are limited studies on the absorption peak characteristics of cellulose in the low frequency band. With the development of science and technology, it is possible to detect the absorption peak characteristics of cellulose by the terahertz spectroscopy technology [7].

Terahertz wave is an electromagnetic wave between microwave and infrared wave with a frequency range of 0.1–10 THz [8]. Terahertz radiation has good penetrability to many dielectric materials and non-polar liquids. In applications of imaging, its nature short wavelength allows to have a longer depth of field while maintaining the same spatial resolution. The image is clearer than X-rays [9]. The terahertz wave photon has low energy, and therefore will not cause damage to the fiber structure and activity. It also has good penetrability to most non-polar materials, so it can be widely used in non-destructive testing and the examination of fiber material properties [10,11]. At the same time, the terahertz spectroscopy technology can also identify and distinguish different configurations and conformations of fibers. These capabilities make the terahertz spectroscopy technology broad applications in the field of fiber materials [12,13].

In recent years, researchers have begun to use the terahertz time-domain spectroscopy technology to study different fiber materials at low frequencies [14,15]. The research on corn, wheat, shell, and reed in the terahertz frequency spectrum characteristics was at 0.2–1.8 THz. The results showed that the using of the terahertz wave technology for plant cellulose detection could achieve quick judgment of cellulose in biomass materials [16,17,18]. Luo et al. [19] calculated 0.2–1.0 THz spectrum absorption coefficient and refractive index. It was showed that the terahertz time-domain spectroscopy technology could be used to detect structural information and vibration of molecules, and applied to identify fibers [19]. Nezadal et al. [20] used a low terahertz frequency band (frequency range of 220–325 GHz) to detect manmade defects in fiberglass reinforced plastics, which can reach a longitudinal resolution of 0.8 mm.

In this study, the terahertz time-domain spectroscopy technology was used to evaluate the crystallinity and crystal structure of cellulose [21,22,23], and to compare with two traditional analysis methods of XRD and FT-IR. It was focused on the terahertz absorption peak characteristics of cellulose at low frequency bands in the range of 0.2–3.0 THz, providing a theoretical basis for the application of fiber materials [24]. The results showed that the crystallinity of cellulose from theory calculations were different between the terahertz spectroscopy detection and traditional analysis [25], while the terahertz spectroscopy detection showed more clearly and intuitively crystallinity characteristics. The absorption peak of the terahertz spectra was the most apparent when the samples’ thickness and ratio were specific. Ultimately, the absorption coefficient decreased with the increase of the sample thickness. At the same time, the degree of crystallinity was proportional to the terahertz absorption coefficient of cellulose, and five movement models of cellulose molecules corresponded to the five absorption peak positions of cellulose studied [26,27].

## 2. Materials and Methods

### 2.1. Materials

Four different scales of cellulose were analyzed in this study. The wood cellulose (WC) samples in this study were provided by KC Flock., Ltd. (Tokyo, Japan), and the microcrystalline cellulose (MCC) samples with the average particle size of 50 μm were provided by Anhui Sunvo Pharmaceutical Materials Co., Ltd. (Anhui, China). The wood cellulose nanofiber (W-CNF) samples were made in laboratory and the cotton cellulose nanofiber (C-CNF) samples with the average diameter of 400 nm and the length of 33 nm were provided by Guilin Qihong technology CO., Ltd. (Guilin, China).

### 2.2. Preparation of Samples

WC and MCC were powder samples and purified to pure cellulose, which could directly be pressed through the tablet press before examinations. W-CNF and C-CNF were prepared to cellulose membrane by freeze-drying. In addition, during the terahertz test, PE (Polyethylene) powder had no characteristic absorption in the terahertz band, which could well reduce the strong absorption effect caused by excessive cellulose sample content. The content of PE was 25 mg, 50 mg, 150 mg, and 250 mg.

### 2.3. Analysis of Crystallinity and Crystal Structure

**Figure 1:**
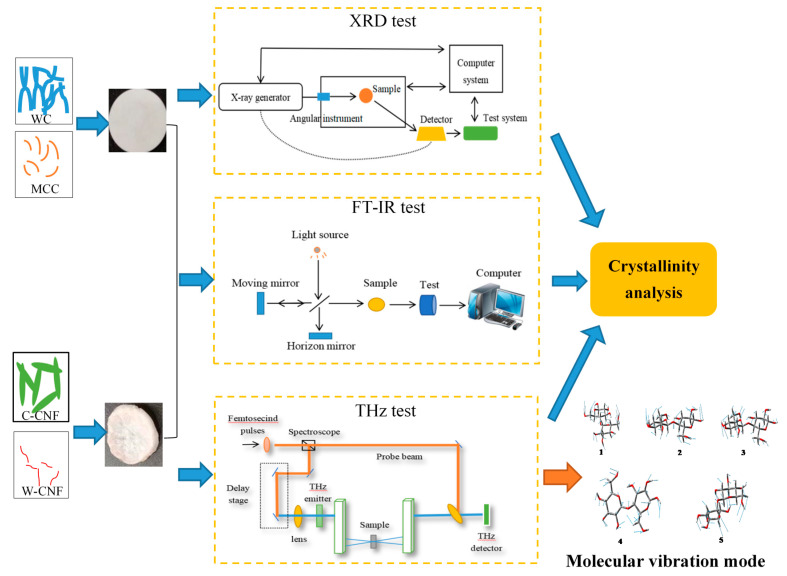
Schematic illustration of description of the entire process.

As shown in Figure 1, the crystallinities of WC, MCC, C-CNF, and W-CNF samples were determined by XRD, FT-IR, and THz. In order to highlight the advantages of the THz method and the area of crystalline region appeared, the location of absorption peak, and the molecular vibration model were established by the density functional theory, showing the types of molecular vibrations caused the changes of absorption peak. The thickness of the sample and the amount of PE added had a certain influence on the transmittance of Terahertz light, and three different thickness samples were selected in the experiment. The addition of the MCC Terahertz spectrum analysis could better explore the absorption characteristics of different cellulose species in the terahertz band. To analyze the absorption characteristics of cellulose in the terahertz frequency band, the vibration model of the molecular structure calculated through the density functional theory, and the characteristic peaks of terahertz absorption were discussed as shown in Figure 1.

#### 2.3.1. Microtopography Analysis

The crystallization characters of WC, MCC, W-CNF, and C-CNF were differently attributed to the different morphologies of samples. To explore the crystallinity, the microscopic morphology characteristics firstly observed by SEM (EM30PLUS, Daejeon, Korea), with a maximum resolution of 5 nm, a magnification factor of 150,000×, and an acceleration voltage of 1~30 kV. Since WC and MCC were powder samples, a point of sample could directly select for the SEM observation. Better images of fiber morphological characteristics could be obtained by constantly adjusting the observation multiple times.

#### 2.3.2. XRD Analysis

The X ray diffractometer (Ultima IV, Rigaku, Japan) was used to determine the crystallinity of cellulose. WC and MCC samples were tableted by the pressing machine. In addition, W-CNF and C-CNF samples were freeze-dried by a freezer dryer (ALpha 1–2 LDplus, Beijing, China) with −40 °C for 12 h. All samples used a Cu Kα radiation in angular range (2θ) from 5° to 50° with a potential of 40 kV and a current of 30 mA, and a scanning speed of 10°/min.

#### 2.3.3. FT-IR Analysis

In this research, the FT-IR test range was 4000–650 cm^−1^ and the resolution was 4 cm^−1^. They were scanned 16 times. The flaking samples made of four kinds of cellulose were placed into the infrared spectrum test bench and the computer collected the effective signals. The noises reduction and horizontal processing were carried out. The absorption peak value of the obtained spectrometer was determined, and the infrared spectrometer of four kinds of cellulose samples were compared. The crystallinity of the absorption peaks was calculated by substituting the values of the absorption peaks using the Equations (4) and (5).

#### 2.3.4. Terahertz Spectroscopy Analysis

The absorption peak characteristics of different cellulose samples were investigated by the THz time-domain spectroscopy (TAS7500SP, Advantest, Japan) [28,29,30,31]. To ensure smooth surface and target thickness, using the tablet press, 10 MPa pressure was applied on all samples for tests. The samples were made of sheets and placed on the terahertz test plate. After the vacuum processing, the interference was removed from the cellulose test in the terahertz band spectrum [32,33,34,35]. Because the absorption intensity of pure cellulose samples might be larger, an appropriate preparation of samples with different thicknesses and different PE blending ratios were discussed. In order to find the optimum parameters of the sample preparation, all samples were made into sheets of a thickness of 0.8 mm, 1 mm, or 1.5 mm, respectively, to evaluate the effect of thickness on the THz time-domain spectroscopy analysis. The samples also mixed with polyethylene for the THz time-domain spectroscopy analysis according to previous research [11], and the mass ratios of different scales of cellulose and polyethylene were 1:1, 1:2, 1:3, and 1:4, respectively.

#### 2.3.5. Molecular Vibration Model

The vibration of molecules in cellulose caused the absorption peak obtained by the terahertz test [36], and the vibration mode of different absorption peak positions was different. To explore the vibration mode of absorption peak at different positions, the molecular structure and crystal cell of cellulose disaccharides calculated by the method using the density functional theory. The corresponding molecular vibration modes at different positions of cellulose were analyzed; thus, the reasons for the variation of absorption intensity in the crystal region was proposed in the next section.

## 3. Results and Discussion

### 3.1. Microtopography

**Figure 2:**
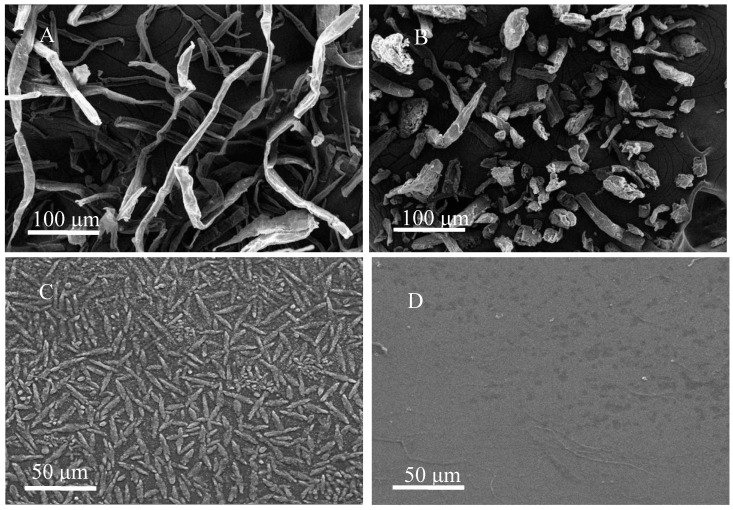
Cellulose samples micromorphology. (**A**) wood cellulose (WC), (**B**) microcrystalline cellulose (MCC), (**C**) cotton cellulose nanofiber (C-CNF), (**D**) wood cellulose nanofiber (W-CNF).

The electron microscope analyses of WC, MCC, W-CNF, and C-CNF are shown in Figure 2. The average length of WC single fiber was 300 μm and diameter was almost around 20 μm. Nevertheless, the shapes of MCC was bar like and the average length was 20 μm. C-CNF was single fibrous substance with a length of 50 μm and a diameter of 25 μm. The average fibrous length of W-CNF was 250 μm and diameter was 6 μm. Cellulose of different scales and dispersity are conspicuously shown in Figure 2, and this could be directly related to the crystallinity and crystal structure of cellulose.

### 3.2. Crystallinity from XRD Analysis.

**Figure 3:**
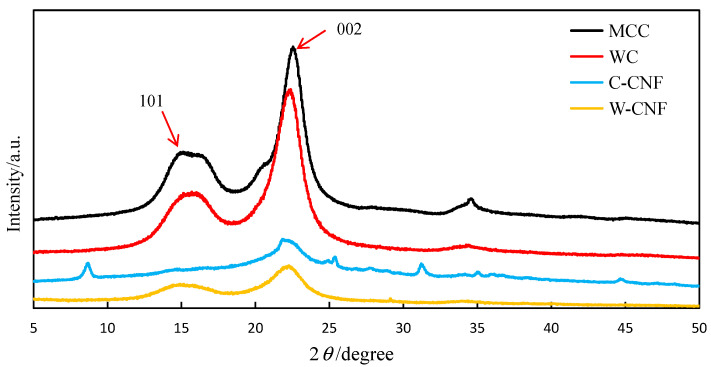
XRD of different cellulose samples.

The crystallinity of all samples tested by the XRD technology, and the results are shown in Figure 3. XRD was the most commonly used method for the determination of the crystallinity of cellulose because it was direct and convenient for the determination of crystallinity. This method could inductively analyze the signal of cellulose crystal structure. In the determination of a cellulose sample, the crystallinity of the cellulose measured by the strength position of the strongest point of the diffraction. There were three different data processing methods for the measured results, namely, three different calculation methods.

The first method was an empirical method proposed by Segal [7] for quickly determining the crystallinity by the XRD pattern. It was also the most used calculation method, namely, the so-called peak strength method. The relative strength of the diffraction peak at the corresponding position on the graph was calculated. The calculation formula was as follows:

(1) $$Crystalline(X_{c})=(I_{002}-I_{am})/I_{002}\times 100\%$$

where *I*_002_ is for 002 crystal plane diffraction intensity, *I*_am_ is for diffraction intensity in the amorphous region, in which, *I*_am_ for 2θ = 18.0° diffraction intensity according to the experience.

The second method was to assume that the sample had a two-phase structure, and the subjective crystalline phase and non-crystalline phase could separate between the minimum diffraction intensity. The calculation formula is presented as follows:

(2) $$Crystalline(X_{c})=S_{Zone}/S_{total}\times 100\%$$

The third method was the peak-splitting method. Lorentz function used for peak splitting of the diffraction curve of the spectrum. The crystallinity calculation formula is described as follows:

(3) $$Crystalline(X_{C})=\left[1-IA/(IA+SP)\right]\times 100\%$$

where *IA* is the integral area of the crystallizing region and *SP* is the total integral area of the crystallizing peak.

In this study, the crystallinity of different scale cellulose samples was calculated by the XRD analysis according to Equation (1). The crystallinity of WC, MCC, W-CNF, or C-CNF was 64.5%, 71.6%, 76.0%, or 76.5%, respectively. It was found that the crystallinity of C-CNF was higher than that of W-CNF due to the higher crystal structure of cotton [16,17], which were consistent with the results of SEM analysis in Section 3.1. The crystallinity of W-CNF was higher than that of MCC, and the crystallinity of MCC was higher than that of WC [18,19]. Different calculation methods of these three could result in different crystallinity calculation results, and perfect single crystal was extremely difficult to obtain. Therefore, XRD was only a relative crystallinity measurement method. In additional, the crystal degree calculated by the XRD method was the ratio of the peak area of a certain main strength peak to the diffuse scattering area of the amorphous region. However, the actual area of the main strength peak also had the contribution of the amorphous region, such as the overlap of the crystalline region and the amorphous region. Moreover, the residual excess substances would cause interference to the results. Therefore, the crystallinity of cellulose measured by the X-ray diffraction method was generally a relative value, which was not accurate enough. More accurate evaluation methods of crystallinity and crystal structure need to be sought.

### 3.3. Crystallinity from FT-IR Analysis

FT-IR analysis was also a common method used to test crystallinity, the Nelson and O’Connord methods were commonly used in the determination of crystallinity of cellulose by FT-IR [37].

When O’Connord [38] was grinding cellulose, it was found that the spectral band at 1429 cm^−1^ of cellulose continued to be weaken, while 893 cm^−1^ continued to be strengthen. Hence the idea of using crystal index O’ki to represent the crystallinity of cellulose. However, this calculation method was only applicable to cellulose. The calculation method is described as follows:

(4) $$O^{\prime }KI=a1429cm^{-1}/a894cm^{-1}$$

Nelson and O’Connord further found that the bending vibration of C-H bond and C-H_2_ bond at 1372 cm^−1^ and 2900 cm^−1^ could also represent the crystallinity index [37], which was calculated using the following equation:

(5) $$N_{.}O^{\prime }KI=a1372cm^{-1}/a2900cm^{-1}$$

In addition, it was found that crystallinity calculated by O’ki had a linear relationship with crystallinity calculated by XRD, and the crystallinity calculated by O’ki had a conic relationship (parabola) with the crystallinity calculated by XRD.

**Figure 4:**
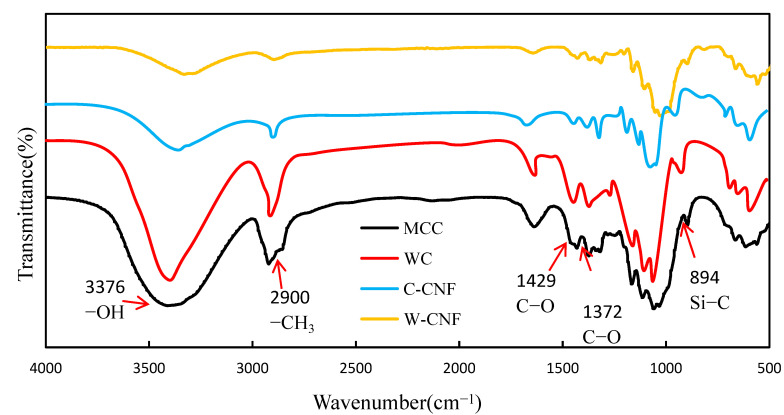
FT-IR of different cellulose samples.

As shown in Figure 4, the crystallinity characteristics were calculated by the FT-IR analysis. The FT-IR method demonstrated that the crystallinity changed according to the change of chemical functional groups. Based on Equations (4) and (5), the crystallinity of MCC was 82%, and its crystallinity calculation result was the lowest. The crystallinity of WC could achieved 92%. The crystallinity of C-CNF was highest. Their crystallinity variation law was consistent with the results from XRD.

Due to the different test bands, the characteristic peak positions of the cellulose performance were different. The crystallinity test results according to FT-IR were different from the results by the XRD analysis. However, their changing trends were the same. The result of the FT-IR analysis also revealed that C-CNF had highest crystallinity comparing to other samples. The crystallinity closely related to the size and structure of the material. From the above results, the XRD and FT-IR methods for analyzing crystallinity both have sharp error value. To estimate the crystallinity more accurately, this study focused on the novel THz time-domain spectroscopy technology for crystallinity and crystal structure analysis.

### 3.4. Crystallinity from THz Analysis

Compared with the lower strength XRD peaking method and FT-IR spectroscopy, the THz time-domain spectroscopy technology was more sensitive to the crystal region. During the test, the crystal region of different types of cellulose would produce sharp absorption peak in THz band, so it was more obvious to the crystal microstructure information. If the crystal structure of cellulose was obvious, the THz spectrometric method was more accurate. Therefore, the terahertz spectrum had a great potential for the crystallization test of cellulose. Based on the model proposed by Dorney et al. [39], the macroscopic optical quality of the sample could be described by the complex refractive index, as follows:

(6) n=n−ik

where *n* is the real refractive index, which is used to describe the dispersion characteristics of samples. *K* is the extinction coefficient used to describe the absorption characteristics of samples.

The relationship between absorption coefficient and extinction coefficient is as follows:

(7) $$\alpha =\frac{2\omega k}{c}$$

where α is the absorption coefficient. C is the speed of light, 3 × 10^8^ m/s. *ω* is angular velocity.

It was apparently shown that the crystallinity resulted from the XRD and FT-IR analysis methods, were different, which was due to the different test methods with some errors existed, including formula and equipment parameters. Therefore, it is necessary to seek a fast and accurate method to obtain the crystallinity of cellulose. There was no doubt that the TH_Z_ spectroscopy was particularly a good choice.

**Figure 5:**
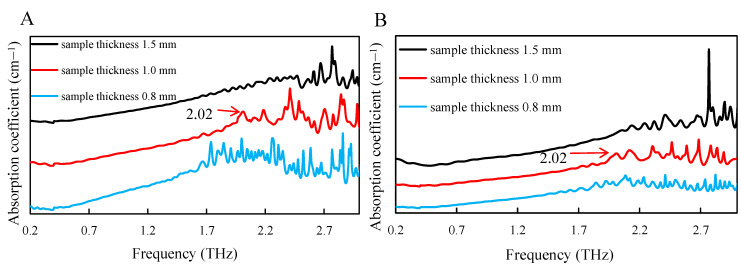
THz analysis of different thickness cellulose samples. (**A**) WC and (**B**) MCC.

To study the crystallinity of cellulose by the THz time-domain spectroscopy technology, the analysis in the 0.2~3.0 THz band was carried out using the terahertz spectroscopy and the test results are shown in Figure 5. According to different attempts, the thickness of the sample would influence the test results of the THz time-domain spectroscopy technology. With the increase of thickness of the sample, the intensity of the absorption peak was smaller, which made the beam could not penetrate samples and obtain the characteristic absorption peak of cellulose samples at this time. When the sample thickness was 1 mm, which could achieve good absorption peak.

When samples were too thick, THz spectral transmittances were low, and the obtained optical absorption rates were unstable. Moreover, there was no great interference when the frequency band was below 1.7 THz, so the absorption characteristics could not be obtained. Due to the high absorption intensity of the THz spectrum, the characteristics of the absorption peak obtained were not obvious. Therefore, the mixture of sample power and polyethylene (PE) could effectively reduce the absorption intensity.

**Figure 6:**
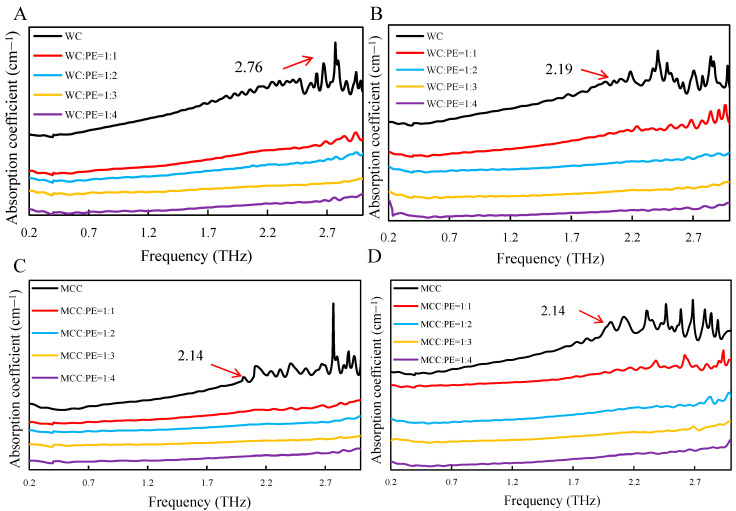
THz test of samples with different mass ratio. (**A**) WC with 0.8 mm thickness, (**B**) WC with 1 mm thickness, (**C**) MCC with 0.8 mm thickness, and (**D**) MCC with 1 mm thickness.

It could be seen in Figure 6 that the absorption strength of the pure sample was the highest. When the mixing ratio of polyethylene increased, the absorption coefficient gradually decreased. Nevertheless, with the increase of the sample thickness, the absorption coefficient continuously reduced. When the mixing ratio of sample with PE was 1:1, the position of the absorption peak was most obvious. The absorption peak shifted toward high frequency, and the mixing ratio of polyethylene also affected the apparent degree of the absorption peak along with the increased thickness. Addition of a small amount of PE could not reduce the absorption strength of cellulose to an appropriate level. However, too much added PE would result in the absorption peak disappeared. Therefore, the proper sample preparation process was an important prerequisite for the accurate measurement of cellulose terahertz band characteristics.

**Figure 7:**
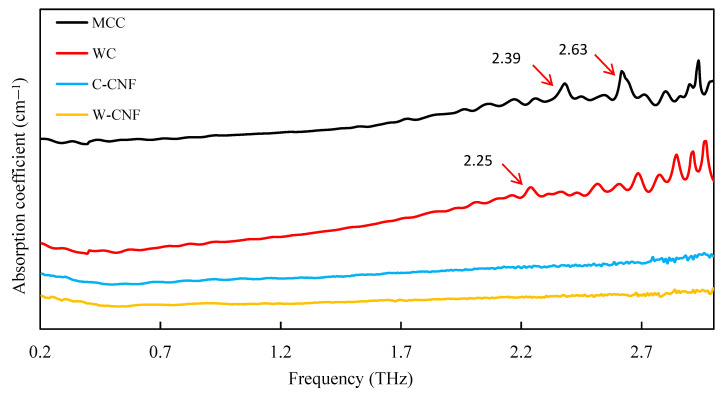
THz test of different cellulose samples.

Based on the previous results, the optimal sample preparation parameters were selected. The optimized mass ratio of sample and polyethylene was 1:1, and the appropriate thickness of the sample was 1 mm. The advantages of the THz spectroscopy in the determination of cellulose crystallinity were analyzed by comparing the absorption peak positions of the four different cellulose samples. The characteristic terahertz absorption peaks of WC, MCC, W-CNF, and C-CNF are shown in Figure 7.

The crystallization of the four kinds of cellulose samples was analyzed by using terahertz spectrum technology, and the crystallinity of terahertz spectrum was calculated, as same as Section 3.2, by using the Equation (2), where *S_Zone_* was the area of the crystallization region, and the S_total_ was total area.

Therefore, the crystallinity of the four cellulose samples under this condition was calculated, and the results were compared with XRD and FT-IR, as shown in the Table 1. Through the test of Terahertz spectrum, it could be found that the absorption peak changes of these four cellulose samples have some differences, which could be used to distinguish them. The crystallinity of cellulose samples in THZ was calculated as follows, 73% for WC, 78% for MCC, 85% for W-CNF, and 90% for C-CNF. Crystallinity obtained by these three methods, their size was consistent with the change, followed by WC, MCC, W-CNF, and C-CNF, and the resulted of the THZ and FT-IR test results, which know the crystallinity of terahertz spectrum test was feasible, in rapidly identify at the same time, could also be calculated on the crystallinity.

### 3.5. Calculation of Cellulose Molecular Vibration Model

**Figure 8:**
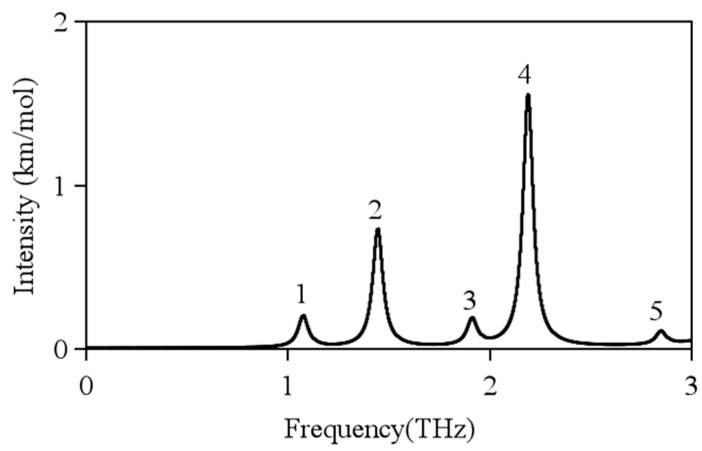
Calculated THz peaks of cellulose in the 0–3 THz frequency range. Lorentzian line shapes convolved into all modes to provide a visual guide.

**Figure 9:**
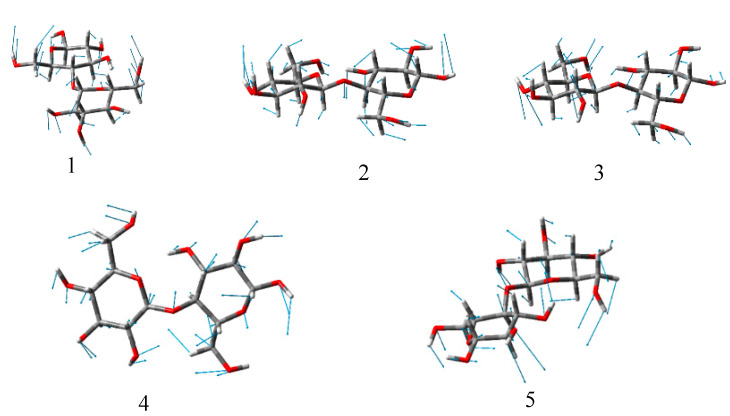
Schematic representation of the atomic motions in the five simulated THz modes of cellobiose.

To understand the different THz absorption features of the four samples examined in this work, the vibrational density of states was reproduced in the low-frequency range and all the infrared active THz modes in these systems. At the current stage, this task was, however, formidable to be achieve at the ab initio level. For having a preliminary trial towards the final solution, the low-frequency vibrations of the building unit, cellobiose, of cellulose were explicitly examined [35,36,39]. The calculation was performed using a combination of the B3LYP functional and the Gaussian-type 6–31G (d, p) basis set in the Gaussian16 software package. Firstly, the geometry of cellobiose in vacuum was optimized and then its vibrational modes and infrared intensity were calculated under the harmonic assumption of the potential surface. The calculation results are displayed in Figure 8. Cellobiose was exhibited in the 0–3 THz frequency range and five optical vibrational modes were designated as #1, #2, #3, #4, and #5 in sequence. Modes #2 and #4 had significantly stronger infrared intensities than all other modes. As shown in Figure 9, both two models featured the skeletal torsional motions between the two glucose segments. Compared with the observation in Figure 7, Modes #2 and #4 might be corresponded to the two fingerprint peaks of MCC around 2.39 THz and 2.63 THz, respectively. If this assignment is correct, the two-fingerprint peaked of MCC would also feature the relative torsions of the glucose segments of the backbones. In this situation, all the glucose united in the backbones vibrated collectively and exhibited a collective wave-like feature [40,41]. This explanation might also apply to the assignment of the small peak around 2.25 THz of WC in Figure 7.

## 4. Conclusions

This research mainly adopted four kinds of cellulose samples, i.e., WC, MCC, W-CNF, and C-CNF. Firstly, SEM was used to analyze their microstructure. The crystallinity of C-CNF was higher than that of W-CNF, the crystallinity of W-CNF was higher than that of MCC, and the crystallinity of MCC was higher than that of WC. Therefore, the Terahertz time-domain spectroscopy had carried on the exploration to the absorption peak and calculated the cellulose structure by the density functional theory. Samples of pure cellulose in terahertz spectrum test, bigger to the interference of the absorption peak, unable to draw samples in terahertz spectrum characteristic absorption peak. With the joining of PE content increasing, samples could be found effectively reduced the absorption intensity, and the most obvious absorption peak appeared when the mixture ratio was 1:1 with polyethylene. Through experiments found that the absorption coefficient of cellulose samples was proportional to the crystallinity, and the absorption peak was formed by the interaction between molecules. There were five vibration modes by the method of modeling.

## Figures and Tables

**Table 1:** Calculation results of crystallinity of four cellulose samples.

Sample	Crystallinity		
	XRD	FT-IR	THz
WC	64%	79%	73%
MCC	72%	82%	78%
W-CNF	76%	97%	85%
C-CNF	77%	98%	90%
